# On the challenge of reconstructing level-1 phylogenetic networks from triplets and clusters

**DOI:** 10.1007/s00285-016-1068-3

**Published:** 2016-10-31

**Authors:** Philippe Gambette, K. T. Huber, S. Kelk

**Affiliations:** 1 LIGM (UMR 8049), CNRS, ENPC, ESIEE Paris, Université Paris-Est, Marne-la-Vallée, 77454 Paris, France; 2 School of Computing Sciences, University of East Anglia, Norwich Research Park, Norwich, NR4 7TJ UK; 3 Department of Knowledge Engineering (DKE), Maastricht University, Maastricht, The Netherlands

**Keywords:** O5C05, 92D15

## Abstract

Phylogenetic networks have gained prominence over the years due to their ability to represent complex non-treelike evolutionary events such as recombination or hybridization. Popular combinatorial objects used to construct them are triplet systems and cluster systems, the motivation being that any network *N* induces a triplet system $\mathcal R(N)$ and a softwired cluster system $\mathcal S(N)$. Since in real-world studies it cannot be guaranteed that all triplets/softwired clusters induced by a network are available, it is of particular interest to understand whether subsets of $\mathcal R(N)$ or $\mathcal S(N)$ allow one to uniquely reconstruct the underlying network *N*. Here we show that even within the highly restricted yet biologically interesting space of level-1 phylogenetic networks it is not always possible to uniquely reconstruct a level-1 network *N*, even when all triplets in $\mathcal R(N)$ or all clusters in $\mathcal S(N)$ are available. On the positive side, we introduce a reasonably large subclass of level-1 networks the members of which are uniquely determined by their induced triplet/softwired cluster systems. Along the way, we also establish various enumerative results, both positive and negative, including results which show that certain special subclasses of level-1 networks *N* can be uniquely reconstructed from proper subsets of $\mathcal R(N)$ and $\mathcal S(N)$. We anticipate these results to be of use in the design of algorithms for phylogenetic network inference.

## Introduction

Phylogenetic trees are essentially graph-theoretical trees whose set of leaves is labelled by a set of species or organisms (more abstractly, *taxa*) and which do not have any degree-two vertices, except possibly the root. They have been the model of choice for many years for shedding light on the evolutionary past of a set of taxa. However, in cases where the taxa are suspected to have undergone reticulate evolutionary events such as hybridization or recombination, trees have been found to not always be appropriate (Sneath 1975). The need for structures capable of appropriately dealing with such data sets, combined with the fact that different evolutionary processes have given rise to them, has resulted in the introduction of a number of more general structures for representing evolutionary relationships. Subsumed under the name “phylogenetic network” these include hybrid phylogenies (Baroni et al. 2006), ancestral recombination graphs (Hein 1990), galled trees (Gusfield et al. 2004; Wang et al. 2001), normal networks (Wilson 2010), regular networks (Baroni et al. 2004), tree-sibling networks (Cardona et al. 2008), level-*k* networks (Jansson et al. 2006; Iersel et al. 2009), median networks (Bandelt 1994) and NeighborNets (Bryant and Moulton 2003), to name just a few, which all generalize a phylogenetic tree in one way or another.

**Fig. 1:**
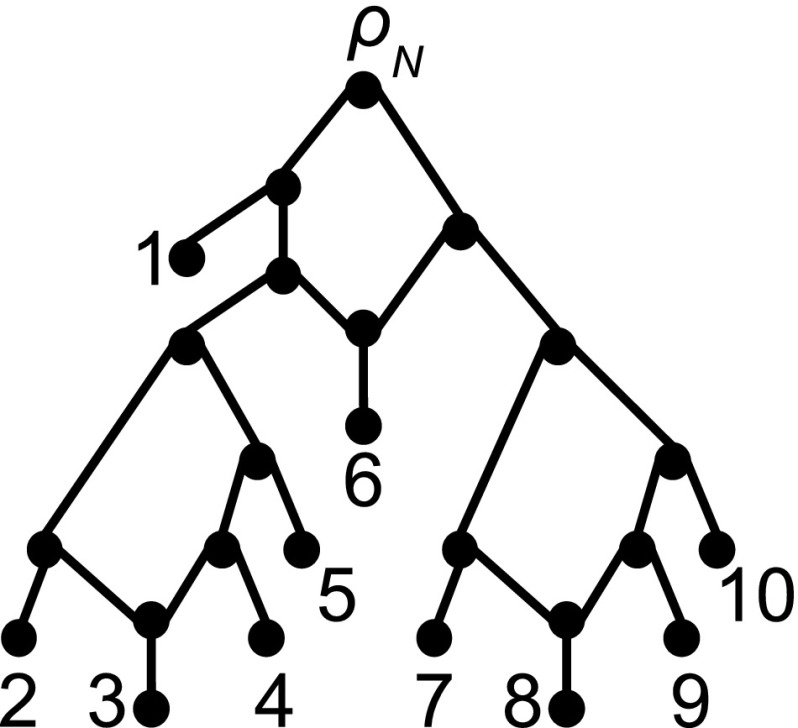
A binary level-1 phylogenetic network *N* on $X=\{1,\ldots , 10\}$ that is also 4-outwards and saturated. As in all figures all arcs of the network are directed downwards, so we do not explicitly indicate the direction of arcs

Apart from median networks and NeighborNets which are a special type of split-based phylogenetic network, the basic graph-theoretical structure underpinning a phylogenetic network is a rooted directed acyclic graph (DAG) that has a unique root and whose set of sinks is a given set of taxa. One of the combinatorially simplest types of phylogenetic network, but still complicated enough to be of interest to Evolutionary Biology, is that of a binary level-1 network (see Fig. 1 for an example). Such structures have attracted a considerable amount of interest in the literature (see e. g. Jansson et al. 2006; Gusfield et al. 2004; Rosselló and Valiente 2009; Huber et al. 2011) and can informally be thought of as degree-constrained rooted DAGs with vertex-disjoint undirected cycles. (Formal definitions of all terms will follow in later sections). However, this simplicity has proven to be deceptive, as the combinatorial structure of such networks has turned out to be more complicated than originally thought (see e.g. Gambette and Huber 2012; Huber and Moulton 2013). Limits on our ability to reconstruct level-1 networks constitute lower bounds on how well we can reconstruct phylogenetic networks more generally. On the other hand, positive results for reconstructing level-1 networks can be an important first step towards algorithms for reconstructing more complex phylogenetic networks.

In this paper, we start by establishing a number of enumerative results for binary level-1 networks. These include upper and lower bounds on the number of vertices and arcs in such networks. We gradually shift our focus onto *cluster systems*, that is, collections of non-empty subsets of the leaves, and *triplet systems*, that is, binary phylogenetic trees on just three leaves. Guided by the fact that these systems have been used for reconstructing phylogenetic networks [see e. g. Morrison (2009) and Huson et al. (2010) for recent overviews], we are particularly interested in finding bounds on the minimum size of a triplet system/cluster system required to “uniquely determine” a level-1 network. For trees this question is well understood. Specifically, for a phylogenetic tree *T* on  $n \ge 3$ leaves it is well-known that *T* is uniquely determined by its induced triplet system $\mathcal R(T)$ (leading to an upper bound of ${n\atopwithdelims ()3}$ for such a minimum-sized set) and that $n-2$ carefully chosen triplets from $\mathcal R(T)$ suffice to uniquely reconstruct *T* when *T* is binary [see Theorem 3 of Steel (1992) and its Corollary]. For this case, it is also well-known that *T* is uniquely determined by its induced cluster system $\mathcal C(T)$ and that for a minimum-sized cluster system to uniquely determine *T*, it must have $|\mathcal C(N)|=2n-1$ elements.

As we shall see, the situation is more complicated for binary level-1 networks. Every level-1 network *N* induces a triplet system $\mathcal R(N)$ and a certain cluster system $\mathcal S(N)$ called the *softwired cluster system of* *N* [see Huson and Scornavacca (2011) for background] but their ability to fully capture the topological structure of *N* is not as strong as one might hope. Let us say that a binary level-1 network *N* is *encoded* by its induced triplet system if for every binary level-1 network $N'$ such that $\mathcal R(N')=\mathcal R(N)$, we have $N = N'$. Continuing, we say that a binary level-1 network is *4-outwards* if its underlying graph does not have a cycle of length four or less. It is precisely the 4-outwards binary level-1 networks *N* that are encoded by $\mathcal R(N)$ as well as $\mathcal S(N)$ (Gambette and Huber 2012) (where we define a binary level-1 network to be encoded by its induced softwired cluster system in an analogous way).

Intriguingly, if $\mathcal R(N')=\mathcal R(N)$ is replaced by $\mathcal R(N)\subseteq \mathcal R(N')$ (as is the case in our formalization of “uniquely determining”) then the assumption that *N* is 4-outwards is no longer strong enough to guarantee uniqueness. A similar observation holds for $\mathcal S(N)$ (see Sects. 6 and 7 for examples for both cases). However, the situation changes for both if, in addition to being 4-outwards we require that *N* is *saturated*, that is, none of its vertices is incident with more than one cut arc (Theorem 6.3 and Theorem 7.3). Simple networks on $n\ge 4$ leaves are 4-outwards, saturated networks that have precisely one cycle in their underlying graph. We show that at most $2n-1$ carefully chosen triplets suffice to uniquely determine such networks. As the network on four leaves depicted in Fig. 6 indicates, this bound is however not tight because five triplets suffice in that case (which can be checked by a simple case analysis). Given that any binary level-1 network *N* contains at least one triplet for any three of its leaves and so $|\mathcal R(N)|\ge {n\atopwithdelims ()3}$ holds, this suggests that at least for simple phylogenetic networks there is a considerable amount of redundancy in $\mathcal R(N)$ with regards to reconstructing *N* from $\mathcal R(N)$. To establish a similar result for general binary level-1 networks *N* might not be straightforward in view of Proposition 4.2, which suggests that $|\mathcal R(N)|$ is not easily expressible in terms of a natural parameter associated with a phylogenetic network *N*, namely its number of non-trivial cut arcs (see Sect. 3). This is somewhat surprising in view of the close relationship between the triplet system induced by a binary level-1 network *N* and its associated softwired cluster system $\mathcal S(N)$ [see e. g. Gambette and Huber 2012, Proposition 2 and Theorem 1 for details concerning this relationship] because the size of $\mathcal S(N)$ *is* closely related to the number of cut arcs of *N* (Theorem 4.1). As in the case of triplet systems, it is easy to find examples of binary level-1 networks *N* that indicate that there is redundancy in the softwired cluster system induced by *N* with regards to uniquely determining *N*. Again focusing on simple networks *N*, we show that at most *n* carefully chosen (softwired) clusters induced by *N* suffice to uniquely determine *N* (Corollary 7.2). However, we do not know if this bound is sharp.

Given that in phylogenetic analyses one is hardly ever guaranteed to have all triplets/clusters induced by a (as yet unknown) phylogenetic network available, the above observations have profound consequences for phylogenetic network reconstruction. One of the most important ones is that a phylogenetic network reconstructed from a triplet or cluster system need not be the network that gave rise to this system.

The paper is organized as follows. In the next section, we present basic terminology of relevance to this paper, including the definition of a level-*k* network and that of a gall in a level-1 network. In Sect. 3, we define cut arcs and present formulas for counting the number of vertices, arcs, and galls in a binary level-1 network. These results improve on the results in Choy et al. (2005) which imply that the number of vertices in a binary level-1 network on *n* leaves is linear in *n* and that the number of hybrid vertices is at most $n-1$. In Sect. 4.1, we formally define the softwired cluster system $\mathcal S(N)$ induced by a binary level-1 network *N* and establish Theorem 4.1. In Sect. 4.2, we define the triplet system $\mathcal R(N)$ induced by a binary level-1 network *N* and establish Proposition 4.2. In Sect. 5, we establish in Proposition 5.1 a relationship between the triplet system induced by a binary level-1 network *N* and a certain partition of the leaf set of *N* that will be crucial for showing Theorem 6.3. In Sect. 6, we first formalize the notion of “uniquely determining” and then present the aforementioned examples for triplet systems. Starting in that section and continuing in Sect. 7, we investigate saturated, 4-outwards, binary level-1 networks and establish Theorem 6.3 and Theorem 7.3, respectively.

## Definitions and notation

In this section we present only basic definitions and notation to avoid overloading the reader. Concepts such as triplets and (softwired) clusters are formalized in subsequent sections.

Throughout the paper, let *X* denote a finite set of size $n\ge 2$. Also all graphs *G* considered have non-empty finite sets of vertices and edges (or arcs in case *G* is directed) and have no loops or multiple edges (or arcs in case *G* is directed).

Suppose for the following that $G=(V,A)$ is a directed acyclic graph (DAG). If *v* and *w* are vertices of *G* such that there exists an arc *a* from *v* to *w* in *G* then we denote that arc by (*v*, *w*) and refer to *v* as the *tail of* *a*, denoted by *tail*(*a*), and to *w* as the *head of* *a*, denoted by *head*(*a*). Suppose $v\in V$ is a vertex of *G*. Then we denote by *outdeg*(*v*) the out-degree of *v* (i.e. the number of arcs whose tail is incident to *v*) and by *indeg*(*v*) the *in-degree* of *v* (the number of arcs whose head is incident to *v*). The sum of the out-degree and the in-degree of *v* is called the *degree* of *v*, denoted by *deg*(*v*). If $indeg(v)=1$ and $outdeg(v)=0$ then *v* is called a *leaf* of *G*. The set of leaves of *G* is denoted by *L*(*G*). Every vertex in $V-L(G)$ is called an *interior vertex* of *G*. If *G* has a unique vertex $\rho =\rho _G\in V$ with $indeg(\rho )=0$ and $outdeg(\rho )\ge 2$ then $\rho$ is called the *root* of *G* and *G* is called a *rooted DAG*. If *G* is a rooted DAG with leaf set *X* and $G'=(V',A')$ is a further rooted DAG with leaf set *X* then we say that *G* is *equivalent* to $G'$ if there exists a graph isomorphism from *G* to $G'$ that is the identity on *X*.

A *phylogenetic network* *N* *on* *X* is a rooted DAG whose set of leaves is *X*, and every interior vertex *v* of *N* except the root $\rho _N$ is either (i) a *split vertex* of *N*, that is, $indeg(v)=1$ and $outdeg(v)\ge 2$ or (ii) a *hybrid vertex* of *N*, that is, $indeg(v)\ge 2$ and $outdeg(v)\ge 1$. In case only the size of *X* is of relevance to the discussion then we will simply call *N* a *phylogenetic network on* |*X*| *leaves* and if the set *X* is of no relevance to the discussion then we will simply call a phylogenetic network *N* on *X* a *phylogenetic network*. We denote the set of hybrid vertices of a phylogenetic network *N* by *H*(*N*) and say that *N* is *binary* if the root of *N* as well as every split vertex of *N* has out-degree two and every hybrid vertex of *N* has out-degree one and in-degree 2.

An undirected graph *G* is called *biconnected* if *G* is connected and $G - v$ is connected for all $v \in V(G)$. A maximal biconnected subgraph *H* of *G* is called a *biconnected component* of *G*. (We say that a biconnected component is *non-trivial* if it contains more than one edge.) Let *U*(*N*) be the *underlying graph* of *N* i.e. the undirected graph obtained from *N* by ignoring the orientation of its arcs. We say that a binary phylogenetic network *N* is a *level-* *k* *(phylogenetic) network*, if every biconnected component of *U*(*N*) contains at most *k* hybrid vertices. Reflecting the fact that a cycle of length three in the underlying graph of a phylogenetic network is indistinguishable (from a triplet or cluster perspective) from a split vertex, we follow common practice and will always assume that a cycle in the underlying graph of a level-1 network *N* contains at least four vertices.

Note that a phylogenetic network *N* for which $H(N)=\emptyset$ holds is simply a *rooted phylogenetic tree* on *X* [sensu Semple and Steel (2003)]. Thus, level-0 networks are rooted phylogenetic trees. All phylogenetic trees considered in this article are rooted so we henceforth drop the “rooted” prefix.

We denote the class of all binary level-1 networks on $n\ge 2$ leaves by ${\mathcal L}_1(n)$. Alternatively, we will also use ${\mathcal L}_1(X)$ to denote that class if we want to emphasize the leaf set *X* of the networks in ${\mathcal L}_1(n)$.

**Fig. 2:**
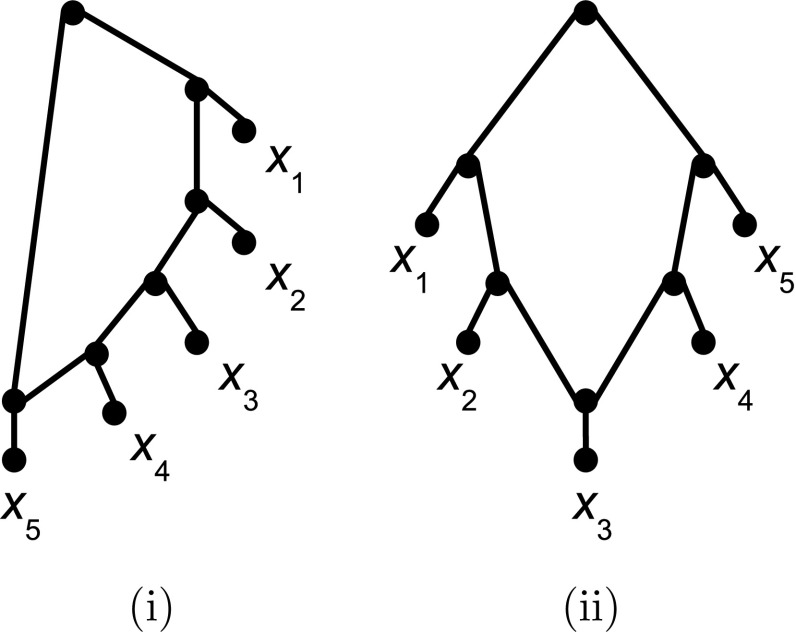
Two examples of simple level-1 networks on $X=\{x_1,\ldots , x_5\}$. Note that both networks are 4-outwards and saturated

Now, suppose that *N* is a level-*k* network, $k\ge 1$. Then we call *N* *proper* if *N* is not also a level-*l* network for some $0\le l\le k-1$. Note that in case $k=1$ such a network must have at least three leaves and at least one hybridization vertex. In that case, we call a non-trivial biconnected component of *U*(*N*) with its original directions in *N* restored a *gall* of *N* and denote the set of galls of a level-1 network *N* by $\mathcal G(N)$. If *N* is binary, contains precisely one gall *C*, and every leaf of *N* is adjacent with a vertex of *C* then *N* is called *simple*. Together with phylogenetic trees, such networks may be viewed as the building blocks of (proper) level-1 networks (Iersel et al. 2009). For the convenience of the reader, we present examples of two simple level-1 networks on $X=\{x_1,\ldots , x_5\}$ in Fig. 2.

## Counting arcs, vertices and galls

In this section, we present some enumerative results concerning the number of vertices, arcs, and galls of a level-1 network. We start by introducing some relevant notation. Suppose *N* is a phylogenetic network on *X*. Following Iersel et al. (2010), we say that a phylogenetic tree *T* on *X* is *displayed* by *N* if there exists a subgraph $N'$ of *N* that is a *subdivision* of *T* i.e. *T* can be obtained from $N'$ by repeatedly suppressing vertices with in-degree and out-degree both equal to 1. For *N* a level-1 network, we denote the number of galls of *N* by *g*(*N*), that is, we let $g(N)=|\mathcal G(N)|$.

### Counting arcs and vertices

In case *N* is a binary level-0 network on $n\ge 2$ leaves, that is, *N* is a binary phylogenetic tree on *n* leaves, it is easy to see that *N* has $2n-1$ vertices and $2(n-1)$ edges (see e. g. Semple and Steel 2003, Proposition 2.1.3 for the corresponding result for unrooted binary phylogenetic trees). For the more general case that *N* is a binary, proper, level-*k* network on $n\ge 2$ (and thus on $n\ge 3$) leaves, and $k\ge 1$, it was shown in (van Iersel 2009, Lemma 4.5) that any such network can contain at most $2n-1+k(n-1)$ vertices and at most $2n-2+\frac{3}{2}k(n-1)$ arcs. Denoting for $n\ge 3$ the subclass of all proper level-1 networks in ${\mathcal L}_1(n)$ by ${\mathcal L}_1(n)^-$, the sizes of the vertex and arc sets of a network $N=(V,A)$ in $\in {\mathcal L}_1(n)^-$ can thus be at most $3n-2$ and $3.5(n-1)$, respectively. Moreover, if follows from (van Iersel 2009, Lemma 4.4) that $|V|=2n+1=|A|$ holds in the special case that *N* is simple. The next result indicates that the size of the vertex set of a simple level-1 network lends itself to providing lower bounds on the sizes of the vertex set and arc set of a general proper level-1 network, respectively.

#### Lemma 3.1

Let $n\ge 3$ and suppose $N = (V,A)\in {\mathcal L}_1(n)^-$. Then $2n + 1 \le |V| \le 3n-2$ and $2n + 1 \le |A| \le 3.5(n-1)$. These bounds are tight if $n=3$, in which case *N* must be a simple level-1 network.

#### Proof

Suppose *X* has size *n* and assume that $N=(V,A)$ is a network in ${\mathcal L}_1(n)^-$ with leaf set *X*. The upper bounds have already been established in the above discussion, so it suffices to prove that $2n+1\le |V|$ and $2n+1\le |A|$. Let $g=g(N)$ and note that $g\ge 1$ holds because *N* is assumed to be proper. We start by adding a new taxon $y \not \in X$ just above the root of *N*, in the following way: introduce a new vertex *u*, add an arc from *u* to the root of *N*, and add an arc from *u* to *y*. Let $N^{y}$ be this new network, whose root is *u*. $N^{y}$ has $|V|+2$ vertices and $|A|+2$ arcs. Let $T = (V_T, A_T)$ be any binary phylogenetic tree on $X \cup \{y\}$ that is displayed by $N^{y}$. Then there exists a subgraph $T'$ of $N^{y}$ that is a subdivision of *T*. Observe that $T'$ must contain 2*g* vertices whose in-degree and out-degree (in $T'$) are both equal to 1. Specifically, *g* of them are hybrid vertices of $N^{y}$ and the other *g* are tails of arcs in $N^{y}$ whose head is contained in $H(N^{y})$. (The correctness of these claims requires the root of $T'$ to be the same vertex as the root of $N^{y}$, and this is the reason for the addition of *y* in the first place.) Consequently, $|V_T|=(|V|+2)-2g$. Noting that *T* has $2(n+1)-1$ vertices (i.e. because it is binary) we have,

$$\begin{aligned} |V| =|V_T|+2g-2 =(2(n+1)-1)+2g - 2 =2n - 1 + 2g \ge 2n+1, \end{aligned}$$

where the last inequality follows because $g \ge 1$. Similarly, to obtain $T'$ from $N^{y}$, we need to delete for every hybrid vertex $h\in H(N^{y})$ precisely one of its incoming arcs (*v*, *h*). Hence, both *v* and *h* will have in-degree and out-degree 1 in $T'$. (Note that *v* might be the root of the gall that contains *h* in $N^{y}$). Hence, $|A_T| = (|A|+2)-g-2g$. Noting (again, because it is binary) that *T* has $2(n+1)-2$ arcs, we have

$$\begin{aligned} |A|=|A_T|+3g - 2 = 2(n+1)-2 + 3g - 2 = 2n + 3g - 2 \ge 2n + 1 \end{aligned}$$

where, as before, the last inequality follows because $g \ge 1$.

It can easily be verified that the bounds are tight in the case $n=3$. Specifically, all expressions evaluate to 7. Finally, if $n=3$ then *N* must be a simple level-1 network, because otherwise it would either be a tree (and thus not proper) or violate the assumption that every cycle in the underlying graph of *N* contains at least four vertices. $\square$

### Counting galls

We next establish a formula for counting the number of galls of a level-1 network. To this end, we require further terminology. Suppose $G=(V,A)$ is a rooted DAG. Then an arc $a \in A$ is called a *cut arc* of *G* if the deletion of *a* disconnects the underlying graph *U*(*G*) of *G*. If *a* is a cut arc of *G* such that *head*(*a*) is a leaf of *G* then we call *a* a *trivial* cut arc of *G* and a *non-trivial* cut arc of *G* otherwise. We denote the number of non-trivial cut arcs of a level-1 network *N* by $c_N$.

Suppose *N* is a level-1 network on *X*. For a gall *C* of *N*, we call an arc of *N* whose tail but not its head is a vertex of *C* an *outgoing arc* of *C*. Note that our assumption that every cycle in *U*(*N*) has at least four vertices implies that every gall must have at least three outgoing arcs. Moreover, if *N* is binary then we call two distinct leaves *x* and *y* of *N* a *cherry* of *N* if *x* and *y* have a common parent. Note that that parent must be a split vertex of *N*. In addition, if *N* is a binary phylogenetic tree and $|X|=3$ then *N* is called a *triplet (on* *X*). Saying that a vertex *v* of a rooted DAG *G* is *below* a vertex *w* of *G* if *w* lies on a directed path from the root of *G* to *v* but is distinct from *v*, we denote a triplet *t* on $X=\{a,b,c\}$ for which the last common ancestor of *a* and *b* is below the root of *t* by *ab*|*c* (or equivalently *c*|*ab*). Finally, a collection $\mathcal R$ of triplets is called a *triplet system (on* $\bigcup _{t\in \mathcal R} L(t)$).

#### Theorem 3.2

Let $n\ge 2$ and suppose $N\in {\mathcal L}_1(n)$. Then $g(N)\le n - c_N - 2$ and this bound is tight if either *N* is a phylogenetic tree or every gall of *N* has exactly three outgoing arcs.

#### Proof

We prove the theorem by induction on $n\ge 2$. Suppose $N\in {\mathcal L}_1(n)$. Then the stated inequality clearly holds in the form of an equality for $n=2$ since in that case *N* is a phylogenetic tree. It also holds for $n=3$ because in that case *N* is either a triplet and so has one non-trivial cut arc but no gall, or *N* is a simple level-1 network and so has precisely one gall but no non-trivial cut arcs.

Suppose that *N* has $$n\ge 4$$ leaves and assume that $$g(N')\le n-1-c_{N'}-2 $$ holds for all level-1 networks $$N'\in {\mathcal L}_1(n-1)$$. Clearly, $$g(N)= n-c_N-2 $$ holds in case *N* is a phylogenetic tree as in that case $$g(N)=0$$ and every non-trivial cut arc of *N* is an interior edge of *N*, of which there are $$n-2$$. So assume that $$N\in {\mathcal L}_1(n)^-$$. To see the stated bound for *g*(*N*), we distinguish between the cases that (i) *N* contains a gall *C* whose outgoing arcs are all trivial cut arcs and (ii) that this is not the case, that is, *N* contains a cherry.

Assume first that Case (i) holds. We distinguish the cases that *C* has three outgoing arcs and that *C* has at least four outgoing arcs. Assume first that *C* has at least four outgoing arcs. Then there must exist a leaf *a* of *N* that is the head of an outgoing arc of *C* but is not adjacent with the unique hybrid vertex of *C*. Consider the rooted DAG $N'$ obtained from *N* by first removing *a*, its parent $a'\in V(N)$, and all arcs adjacent with $a'$ and then adding a new arc from the parent of $a'$ to the child of $a'$ contained in *V*(*C*). Clearly, $N'$ is a binary level-1 network on $L(N){\setminus }\{a\}$ and $g(N)=g(N')$ and $c_N=c_{N'}$ both hold. Since $|L(N')|= n-1$, we have $g(N)=g(N')=n-1 - c_{N'} -2=n-3 - c_N< n-c_N-2$, by the induction hypothesis. Consequently, $g(N)<n - c_N -2$ holds in this case.

Next, assume that *C* has exactly three outgoing arcs $a_1,a_2,a_3$. Let $N'$ be the rooted DAG obtained from *N* by contracting *C* as well as $a_1$, $a_2$, and $a_3$ into a new leaf *x*. Clearly, $N'$ is a binary level-1 network on $L(N)\cup \{x\}{\setminus }\{head(a_1),head(a_2), head(a_3)\}$ and $g(N')=g(N)-1$ and $c_{N'}=c_N-1$. Thus, $g(N') \le n-2 - c_{N'} -2$ and, so, $g(N) \le n-c_N-2$ holds in this case too.

Last but not least, assume that Case (ii) holds, that is, *N* contains two leaves *x* and *y* that form a cherry. Let $N'$ denote the rooted DAG obtained from *N* by first deleting *x*, its parent *p*, and all arcs incident with *p* and then adding a new arc from the parent of *p* to *y*. Clearly $N'$ is a binary level-1 network on $L(N){\setminus }\{x\}$ and $g(N')=g(N)$ and $c_{N'}=c_N-1$ both hold. Consequently, $g(N)=g(N') \le n-1 - c_{N'} -2= n-1-(c_N-1)-2=n-c_N-2$ holds by the induction hypothesis. This concludes the proof of this case and thus the proof of the stated bound for *g*(*N*).

It remains to establish that the stated bound for *g*(*N*) is tight for a level-1 network $N\in {\mathcal L}_1(n)$ for which all of its galls have precisely three outgoing arcs. To see this, one can again perform induction on $n\ge 2$ but this time assuming that $g(N')=n-c_{N'}-2$ holds for all level-1 networks $N'\in {\mathcal L}_1(n-1)$ for which every gall has precisely three outgoing arcs. In this context it should be noted that the cases $n\in \{2,3\}$ and *N* is a phylogenetic tree on *n* leaves have already been observed above. We leave the details to the interested reader. $\square$

## Counting clusters and triplets

In this section we establish enumerative results for computing the sizes of the so-called hardwired and softwired cluster system, respectively, that have both been introduced in the literature for phylogenetic network reconstruction (Huson et al. 2010). In addition, we establish that the corresponding result for triplets does not hold. We start with clusters.

### Counting clusters

We call a non-empty subset of *X* a *cluster* and refer to a set of clusters of *X* as a *cluster system on* *X*, or just a cluster system if the set *X* is clear from the context. Suppose for the following that *N* is a phylogenetic network on *X* and that $v\in V(N)$. Then we define the cluster $C_N(v)$ associated with *v* to comprise of all leaves of *N* that are below *v* and let $C_N(v)=\{v\}$ in case *v* is a leaf of *N*. Again, we simplify our notation by writing *C*(*v*) rather than $C_N(v)$ if *N* is clear from the context. Note that $C(\rho _N)=X$. Then the *hardwired cluster system* $\mathcal C(N)$ associated with *N* is the cluster system $\{C(v)\,:\, v\in V(N)\}$. Note that if $N\in {\mathcal L}_1(X)^-$, then Lemma 3.1 implies that $2n+1\le |\mathcal C(N)|\le 3n-2$ and if *N* is a phylogenetic tree, then $|\mathcal C(N)|=|V(N)|=2n-1$, where *n* denotes |*X*| in both cases. Denoting by $\mathcal T(N)$ the set of phylogenetic trees on *X* displayed by *N*, the *softwired cluster system* $\mathcal S(N)$ associated with *N* is defined as $\mathcal S(N)=\bigcup _{T\in \mathcal T(N)} \mathcal C(T)$.

**Fig. 3:**
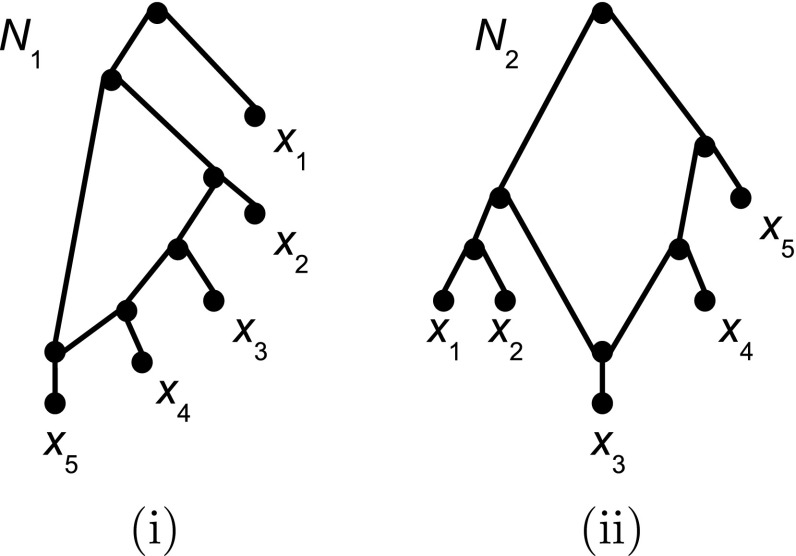
Two networks $N_1$ and $N_2$ on $X=\{x_1,\ldots , x_5\}$. Note that both networks are 4-outwards, but neither is saturated

To illustrate this definition, consider the level-1 network $N_1$ on $X=\{x_1,\ldots ,x_5 \}$ depicted in Fig. 3i. Then $\mathcal S(N_1)$ comprises the clusters *X*, $\{x_2, x_3, x_4, x_5\}$, $\{x_3,x_4,x_5\}$, $\{x_4,x_5\}$, $\{x_2,x_3,x_4\}$, $\{x_3,x_4\}$, $\{x_1\}$, $\{x_2\}$, $\{x_3\}$, $\{x_4\}$, and $\{x_5\}$.

It is not too difficult to argue that $\mathcal S(N)$ contains *O*(*n*) clusters. To see this, let *T* be a tree on *X* displayed by *N* and let *v* be a vertex of *T*. From the definition of display it follows that a subdivision of *T* can be topologically embedded within *N*. Fix such an embedding, and let $T'$ and $v'$ be the images of *T* and *v* in *N*. If $v'$ is the head of a cut arc in *N*, or the root of *N*, then $C_T(v)$ will be equal to $C_{N}(v')$, irrespective of the exact embedding. If *v* is not the head of a cut arc, nor the root, then it is a vertex of some gall of *N*. In that case, there are only (at most) two possibilities for $C_T(v)$. Specifically, the choice of cluster is completely determined by which of the two edges incoming to the hybridization vertex in the gall, are in $T'$ (irrespective of the exact topology of the embedding). Now, from Lemma 3.1 *N* contains *O*(*n*) vertices. Given that (as argued) each vertex can contribute at most two clusters, it follows that $\mathcal S(N)$ contains *O*(*n*) clusters. The next result improves on this *O*(*n*) observation by providing a formula for the size of the closely related cluster system $\mathcal S(N)^-:=\mathcal S(N){\setminus }\{X\}$. (This is also an improvement on the result presented in Gambette and Huber 2012, Proposition 3.) To establish it, we require further terminology.

Suppose $N\in {\mathcal L}_1(X)$ and $X' \subseteq X$. Then we define the *restriction* $N|_{X'}$ *of* *N* *to* $X'$ to be the network in ${\mathcal L}_1(X')$ obtained from *N* by deleting all vertices in $X - X'$ and then applying the following “cleaning up” operations in any order until no more can be applied^1^: (1) suppressing vertices with in-degree and out-degree both equal to one; (2) deleting vertices with out-degree zero that are not an element in *X*; (3) collapsing multi-arcs into a single arc; (4) if a gall *G* has been created that has exactly two outgoing cut arcs (*u*, *v*), $(u',v')$, then deleting these two cut arcs and all the arcs of *G* and adding arcs (*r*, *v*) and $(r, v')$, where *r* is the unique vertex of *G* whose children are *u* and $u'$; (5) deleting vertices with in-degree zero and out-degree one. (Note that if *N* is a phylogenetic tree this definition specializes to the usual definition of “restriction” used in the tree literature.) We often write $N|_{X-x}$ as shorthand for $N|_{X - \{x\}}$.

#### Theorem 4.1

Let $n\ge 2$ and suppose $N\in {\mathcal L}_1(n)$. Then $|\mathcal S(N)^-|=3n - 4 - c_N$.

#### Proof

We prove the theorem by induction on $n\ge 2$. Suppose $N\in {\mathcal L}_1(n)$. Then the stated equality holds if $n=2$ as then *N* is a phylogenetic tree on two leaves and if $n=3$ because in that case *N* is either simple and so $c_N=0$ holds or *N* is a triplet. In the former case, $|\mathcal T(N)|= 2$ and both phylogenetic trees contained in $\mathcal T(N)$ are triplets. Thus, $|\mathcal S(N)^-|=5=3n - 4 - c_N$ holds in this case. In the latter case, $c_N=1$ follows and thus $|\mathcal S(N)^-|=4=3n - 4 - c_N$ in this case, too.

Now suppose $n>3$ and assume that the theorem holds for all networks $N'$ with at most $n-1$ leaves. Let $X=L(N)$. We distinguish between the cases that every cut arc of *N* is trivial and the case that *N* contains at least one non-trivial cut arc.

Suppose first that every cut arc of *N* is trivial. Then $c_N = 0$ and *N* is simple. Note that since $n>3$, at least one of the two directed paths from the root $\rho _N$ to the hybrid vertex $h_N$ of *N* must contain at least two vertices distinct from $\rho _N$ and $h_N$. Let $P_1$ denote such a path. Moreover, let $v\in V(P_1)$ denote the vertex on $P_1$ that is adjacent with $\rho _N$ and let $x\in X$ denote the leaf of *N* that is adjacent with *v*. Let $X'=X-\{x\}$ and $N'=N|_{X'}$. Clearly $N'\in {\mathcal L}_1(n-1)$ and $c_{N'}=c_N=0$. Thus, $|\mathcal S(N')^-|=3(n-1) - 4$, by the induction hypothesis. Observe that the definition of *S*(*N*) implies that $S(N)^{-}$ contains exactly three clusters that $S(N')^{-}$ does not. Indeed, in case the other directed path from $\rho _N$ to $h_N$ also contains vertices distinct from $\rho _N$ and $h_N$, the three clusters missing from $S(N')^{-}$ are $\{x\}$, $C_N(v){\setminus }\{h\}$ and $C_N(v)$, where *h* is the leaf below $h_{N}$. Otherwise, the three clusters missing from $S(N')^{-}$ are $\{x\}$, $C_N(v){\setminus }\{h\}$ and $C_{N}(v')$ where $v'$ is the child of *v* that is not contained in *X*. Consequently, $|\mathcal S(N)^-|=|\mathcal S(N')^-| +3= 3(n-1) - 4-0+3=3n-4-c_N$ holds in this case.

Now suppose that *N* has a non-trivial cut arc $e=(u,v)$. Let $Y_1=\{l\in X: l \text{ is } \text{ below } v\}$ and $Y_2=X-Y_1$. Note that $2\le |Y_1|<n$. Hence, $1\le |Y_2|\le n-2$. Consider the rooted DAG $N_1$ with leaf set $Y_1$ obtained from *N* by deleting all vertices (plus their incident arcs) that are not below *v* and the rooted DAG $N_2$ on $Y_2\cup \{v\}$ obtained from *N* by deleting all vertices below *v* (plus their incident arcs). Since $|Y_1|\ge 2$ it follows that $N_1\in {\mathcal L}_1(Y_1)$ and since $|Y_2\cup \{v\}|\ge 2$, we have that $N_2\in {\mathcal L}_1(Y_2\cup \{v\})$. Note that a phylogenetic tree *T* is displayed by *N* if and only if $T|_{Y_1}$ is displayed by $N_1$ and $T|_{Y_2\cup \{v\}}$ is displayed by $N_2$. Consequently, $\mathcal S(N)^-=\mathcal S(N_1)^- \mathop {\cup }\limits ^{\cdot }\{C\in \mathcal S(N_2)^-:v\not \in C\} \mathop {\cup }\limits ^{\cdot }\{C-\{v\}\cup Y_1:v\in C \text{ and } C\in \mathcal S(N_2)^-\}$ must hold. Thus,

$$\begin{aligned} |\mathcal S(N)^-|=|\mathcal S(N_1)^-|+ |\mathcal S(N_2)^-|. \end{aligned}$$

Let $i=1,2$ and let $n_i=|L(N_i)|$ and $c_i=c_{N_i}$. Then $|\mathcal S(N_i)^-|=3n_i - 4 - c_i$, by the induction hypothesis. Consequently, $|\mathcal S(N)^-|=3n_1 - 4 -c_1 + 3n_2 - 4 - c_2$. Since $n_1 + n_2 = n + 1$ and $c_1 + c_2 = c_N - 1$ it follows that $|\mathcal S(N)^-| =3(n + 1) - 8 - (c_N - 1) = 3n - 4 - c_N,$ holds in this case, too. $\square$

### Counting triplets

In view of the close relationship between the cluster system $\mathcal C(T)$ induced by a phylogenetic tree *T* on at least three leaves and the triplet system $\mathcal R(T)$ induced by *T* [see e. g.  (Dress et al. 2012) or (Iersel and Kelk 2011)] it is reasonable to hope that the companion result to Theorem 4.1 might hold for the triplet system $\mathcal R(N)$ induced by a phylogenetic network *N* on at least three leaves. Put differently, it should be possible to express the size of $\mathcal R(N)$ in terms of the number of galls and non-trivial cut arcs of *N*. As the next result shows, this is not the case. We start with defining the triplet system $\mathcal R(N)$.

Suppose $N\in {\mathcal L}_1(X)$, where $|X|\ge 3$ and *a*, *b*, and *c* are distinct elements in *X*. Then the triplet *ab*|*c* is said to be *consistent* with *N* if there exist distinct vertices *v* and *w* in *N* and directed paths in *N* from *v* to *c* and *w*, respectively, and from *w* to *a* and *b*, respectively, such that any pair of those paths does not have an interior vertex in common. The triplet system $\mathcal R(N)$ is then the set of all triplets *t* with $L(t)\subseteq X$ that are consistent with *N*.

To illustrate this definition consider the simple level-1 network $N_2$ on $X=\{x_1,\ldots ,x_5\}$ depicted in Fig. 2ii. Then $\mathcal R(N_2)$ comprises the sixteen triplets $x_3|x_1x_2$, $x_4|x_1x_2$, $x_5|x_1x_2$, $x_1|x_3x_4$, $x_4|x_1x_3$, $x_1|x_3x_5$, $x_5|x_1x_3$, $x_1|x_4x_5$, $x_2|x_3x_4$, $x_4|x_2x_3$, $x_2|x_3x_5$, $x_5|x_2x_3$, $x_2|x_4x_5$, $x_3|x_4x_5$, $x_5|x_3x_4$, $x_1|x_2x_3$.

#### Proposition 4.2

For all $n\ge 6$, there exist distinct networks $N_1,N_2\in {\mathcal L}_1(n)$ with the same number of galls and non-trivial cut arcs but $|\mathcal R(N_1)|\not =|\mathcal R(N_2)|$.

#### Proof

**Fig. 4:**
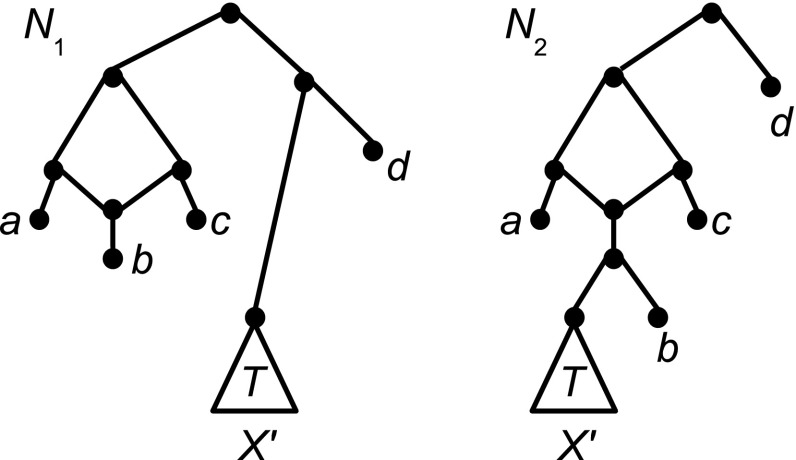
Two binary level-1 networks $N_1$ and $N_2$ on $X'\cup \{a,b,c,d\}$ for which the respective number of leaves, galls, and non-trivial cut arcs are the same yet $|{\mathcal R}(N_2)|\not =|{\mathcal R}(N_1)|$ – see the proof of Proposition 4.2 for details

Let $X'$ denote a finite set of size at least two and let *a*, *b*, *c*, and *d* denote pairwise distinct elements not contained in $X'$. Consider the binary level-1 networks $N_1$ and $N_2$ on $X:=X'\cup \{a,b,c,d\}$ depicted in Fig. 4, where the triangle marked *T* denotes some binary phylogenetic tree on $X'$.

As can be easily checked, $N_1$ and $N_2$ have the same number of leaves and both contain one gall and have $c_{T}+3$ non-trivial cut arcs. Moreover, for any 3-set $Y\in {X\atopwithdelims ()3}$, there exists exactly one triplet on *Y* that is contained in ${\mathcal R}(N_1)$ except for $Y=\{a,b,c\}$ for which $a|bc, c|ab\in {\mathcal R}(N_1)$ holds. Hence, $$|{\mathcal R}(N_1)|=\left( {\begin{array}{c}n\\ 3\end{array}}\right) +1$$, where $n=|X|$. Similarly for every 3-subset $Y\in {X\atopwithdelims ()3}$, there exists exactly one triplet on *Y* that is contained in ${\mathcal R}(N_2)$ except for $Y=\{a,c,x\}$ with $x \in X'\cup \{b\}$ for which $a|cx,c|ax\in {\mathcal R}(N_2)$ holds. Consequently, $$|{\mathcal R}(N_2)|=\left( {\begin{array}{c}n\\ 3\end{array}}\right) +1+|X'|>\left( {\begin{array}{c}n\\ 3\end{array}}\right) +1= |{\mathcal R}(N_1)|$$.

$$\square $$

## Triplet systems and the partition *Cut*(*N*)

In this section, we start turning our attention to the question of how many triplets suffice to uniquely determine a binary level-1 network. Central to our arguments will be a special type of subsets of *X* called SN-sets which were originally introduced in Jansson et al. (2006) and further studied in, for example, Iersel et al. (2009), Iersel and Kelk (2011).

Suppose $|X|\ge 3$ and $\mathcal R$ is a triplet system on *X*. Then a subset $S\subseteq X$ is called an *SN-set* of $\mathcal R$ if there is no triplet $xy|z\in \mathcal R$ with $x, z \in S$ and $y \not \in S$. In addition, such a set *S* is called *non-trivial* if $S\not =X$.^2^ Last but not least, a non-trivial SN-set *S* for $\mathcal R$ is called *maximal* if there is no non-trivial SN-set that is a strict superset of *S*.

As it turns out, for a binary network *N* (of any level) the SN-sets of $\mathcal R(N)$ are closely related to the cut arcs of *N* in the sense that if (*u*, *v*) is a cut arc of *N*, then $C_N(v)$ is an SN-set of $\mathcal R(N)$ because there cannot exist a triplet $xy|z \in \mathcal R(N)$ such that $x,z\in C_N(v)$ and $y\not \in C_N(v)$. We call a cut arc (*u*, *v*) of *N* *highest* if there does not exist a cut arc $(u', v')$ of *N* such that there is a directed path from $v'$ to *u*. We denote by *Cut*(*N*) the partition of *X* induced by, for each highest cut arc (*u*, *v*) of *N*, taking the cluster $C_N(v)$ of *X*. By (Iersel and Kelk 2011, Observation 3) *Cut*(*N*) is exactly the set of maximal SN-sets of $\mathcal R(N)$.

To illustrate, consider the network $N_1$ on $X=X'\cup \{a,b,c,d\}$ depicted in Fig. 4. Then $Cut(N_1)$ is the bipartition $\{\{a,b,c\},X' \cup \{d\}\}$.

We begin with an auxiliary observation which relies on the concept of compatibility of pairs of sets, whereby two non-empty finite sets *A* and *B* are called *compatible* if $A\cap B\in \{\emptyset ,A,B\}$ holds and *incompatible* otherwise. More generally, a cluster system $\mathcal C$ is called *compatible* if any two clusters in $\mathcal C$ are compatible and *incompatible* otherwise (see e. g. Semple and Steel 2003, Section 3.5 and Dress et al. 2012 for more on such objects).

### Observation 1

Suppose that $n\ge 3$ and that *N* and $N'$ are two networks in ${\mathcal L}_1(n)$ such that $\mathcal R(N) \subseteq \mathcal R(N')$. Let $v\in V(N)$ and $v'\in V(N')$ denote two split vertices that are heads of cut arcs of *N* and $N'$, respectively. Then the induced clusters $C_N(v)$ and $C_{N'}(v')$ are compatible. In particular, if $C_{N}(v) \subsetneq C_{N'}(v')$ then $C_N(v)$ is not a maximal SN-set for $\mathcal R(N)$.

### Proof

Let $C=C_N(v)$ and $C'=C_{N'}(v')$. Clearly, if $C=C'$ then *C* and $C'$ are compatible. So suppose $C\not =C'$. Assume for the sake of contradiction that *C* and $C'$ are not compatible, that is, $C\cap C'\not \in \{\emptyset ,C,C'\}$. Choose elements $x \in C{\setminus }C'$, $y \in C \cap C'$ and $z \in C'{\setminus }C$. Then, out of the three possible triplets with leaf set $\{x,y,z\}$, only the triplet *xy*|*z* can be contained in $\mathcal R(N)$. Hence, $xy|z\in \mathcal R(N')$ and, so, $C'$ cannot be an SN-set of $\mathcal R(N')$; a contradiction as the incoming arc of $v'$ is a cut arc of $N'$ and, so, $C'$ must be an SN-set of $\mathcal R(N')$. Thus, *C* and $C'$ must be compatible.

To see the remainder of the observation, assume that $C\subsetneq C'$. Then since $\mathcal R(N) \subseteq \mathcal R(N')$ and $C'$ is an SN-set of $\mathcal R(N')$, it follows that $C'$ is also an SN-set of $\mathcal R(N)$. Since *C* is also an SN-set of $\mathcal R(N)$, it cannot be a maximal SN-set of $\mathcal R(N)$. $\square$

The next result will be required by the induction argument that we will use in the proof of Theorem 6.3. The proof of the proposition relies on the facts that for any saturated network $N\in {\mathcal L}_1(X)$ (i) the partition *Cut*(*N*) contains at least three elements and (ii) there exists a gall *B* of *N* such that the root of *N* is a vertex of *B*.

**Fig. 5:**
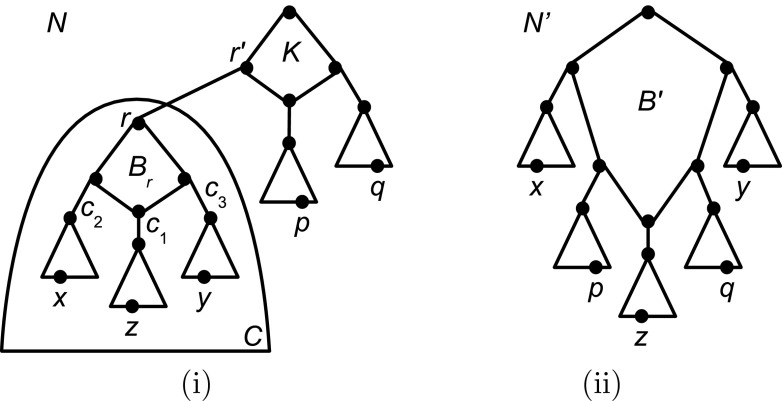
The structure of networks **i** *N* and **ii** $N'$ considered in the proof of Proposition 5.1

### Proposition 5.1

Suppose that $|X|\ge 3$, that *N* is saturated network in ${\mathcal L}_1(X)$ and that $N'$ is a network in ${\mathcal L}_1(X)$ such that $\mathcal R(N) \subseteq \mathcal R(N')$. Then $Cut(N)=Cut(N')$.

### Proof

The proof contains multiple parts so we first describe it at a high level, and then give details. The entire proof is devoted to proving that $Cut(N)\subseteq Cut(N')$, and after this $Cut(N) = Cut(N')$ follows immediately from the fact that *Cut*(*N*) and $Cut(N')$ are both partitions of *X*. The proof that $Cut(N)\subseteq Cut(N')$ holds is a long proof by contradiction, which starts with the assumption that there exists some $C\in Cut(N)-Cut(N')$. We then show that $|C|\ge 2$ must hold. Combined with the assumption that *N* is saturated we then infer that, up to symmetry, the structure of *N* is as indicated in Fig. 5i. Choosing elements $x,p,q\in X$ as described below we obtain that $\mathcal R(N)$ must contain two distinct triplets $t_1$ and $t_2$ with leaf set $\{x,p,q\}$. By examining the structure of $Cut(N')$ we identify that at least one of two cases, referred to as Cases (i) and (ii) below, must hold. However, we show that Case (i) cannot hold, and thus conclude that Case (ii) must hold. We then show that, up to symmetry, the structure of $N'$ is as indicated in Fig. 5ii. We argue that *x*, *p*, *q* are below three distinct highest cut arcs of $N'$, and that none of these are the cut arc incident to the hybridization vertex of $B'$ (where $B'$ is the topmost gall of $N'$, as shown in Fig. 5ii). This implies that $\mathcal R(N')$ cannot contain both $t_1$ and $t_2$ which finally yields the required contradiction.

Let us then start by assuming, for the sake of contradiction, that there exists some $C\in Cut(N)-Cut(N')$.

*Proof that* $|C|\ge 2$: Since both *Cut*(*N*) and $Cut(N')$ are partitions of *X*, there exists some $C'\in Cut(N')$ distinct from *C* such that $C\cap C'\not =\emptyset$. Since, in view of (Iersel and Kelk 2011, Observation 3) recalled above, *C* is a maximal SN-set of $\mathcal R(N)$, and, by Observation 1, *C* and $C'$ are compatible, it follows that $C'\subsetneq C$. Thus, there exists a further element $C''\in Cut(N')$ distinct from *C* and $C'$ such that $C\cap C''\not =\emptyset$ holds, too. Thus, $|C|\ge 2$.

*The structure of* *N*: Let $r\in V(N)$ denote the head of the cut arc $(r',r)$ of *N* for which $C=C_N(r)$ holds and let $B_r$ denote the gall of *N* that contains *r* in its underlying cycle (which exists because $|C|\ge 2$ and *N* is saturated). In view of the usual assumption that no gall in a phylogenetic network has two or fewer outgoing cut arcs, $B_r$ has at least three outgoing cut arcs $c_1, c_2$ and $c_3$ (see Fig. 5i). Let $c_1$ denote the outgoing cut arc of $B_r$ whose tail is the hybrid vertex $h_{B_r}$ of $B_r$. Let $z\in C_N(h_{B_r})$, let $x\in C_N( head(c_2) )$ and let $y\in C_N( head(c_3) )$. Clearly, $\mathcal R(N)$ contains two distinct triplets *t* and $t'$ on $\{x,y,z\}$.

*The structure of* $N'$: Since $\mathcal R(N)\subseteq \mathcal R(N')$ we also have $t,t'\in \mathcal R(N')$. Since $Cut(N')$ is the partition of *X* induced by the maximal SN-sets of $\mathcal R(N')$, it follows that either (i) there exists some element $A\in Cut(N')$ such that $x,y,z\in A$ or (ii) there exist distinct elements $C_x,C_y,C_z\in Cut(N')$ such that $a\in C_a$, for all $a\in \{x,y,z\}$.

Assume first that Case (i) holds. We claim that $C \subseteq A$. To see this, note that we were free to choose any two cut arcs $c_2$ and $c_3$ distinct from $c_1$ and subsequently we had a free choice of *z*, *x*, *y*. For any $Z:=\{x,y,z\}$ chosen this way—let us call this a valid choice—it is straightforward to see that there exist two triplets on *Z* in $\mathcal {R}(N)$ and thus in $\mathcal {R}(N')$. Since *A* is an SN-set of $\mathcal {R}(N')$ it follows that as soon as two of the three leaves of a triplet on *Z* are contained in *A*, so too is the third. Now, let $\{x,y,z\}$ be our initial valid choice, so by assumption $\{x,y,z\} \subseteq A$. Simple case analysis shows that for any element $p\in C$, at least one of $\{x,y,p\}$, $\{x,p,z\}$ or $\{p,y,z\}$ is a valid choice. Hence, $p\in A$ which proves the claim. Since $C \not \in Cut(N')$ we have in fact $C\not =A$. But $C\subsetneq A$ cannot hold either because *C* is a maximal SN-set for $\mathcal {R}(N)$ and *A* is a maximal SN-set for $\mathcal {R}(N')$, and by Observation 1 this cannot happen. Thus, Case (ii) must hold (see Fig. 5ii).

*The triplets* $t_1$ *and* $t_2$: Let $h\in V(N)$ denote the hybrid vertex of the topmost gall *K* of *N*, that is, the gall of *N* that contains the root of *N* in its vertex set (which must exist because *N* is saturated). Also note that because $C\in Cut(N)$ it follows that $(r',r)$ is a highest cut arc of *N* and thus $r'$ is a vertex of *K*. Since $|Cut(N)|\ge 3$ there exist distinct elements $C_1,C_2\in Cut(N)-\{C\}$ such that $C_N(h)\in \{C,C_1,C_2\}$. Choose some $p\in C_1$ and some $q\in C_2$. Combined with the definition of $\mathcal R(N)$ it follows that $\mathcal R(N)$ must contain two triplets $t_1$ and $t_2$ on $\{x,p,q\}$, two triplets on $\{y,p,q\}$, and two triplets on $\{z,p,q\}$. Note that since $\mathcal R(N)\subseteq \mathcal R(N')$, those six triplets are also contained in $\mathcal R(N')$. (In the next part of the proof we assume $C_{N'}(h') = C_z$, where these terms will be defined in due course, and the critical point here is that $C_{N'}(h') \ne C_x.$ This is genuinely without loss of generality because when selecting $t_1$ and $t_2$ in the present part of the proof it does not matter whether they are on $\{x,p,q\}$, $\{y,p,q\}$ or $\{z,p,q\}$.)

*The taxa* *x*, *p*, *q* *are all beneath distinct highest cut arcs of* $N'$, *but none of these are incident to the hybridization vertex:* Using *x*, *y*, *z*, *p* and *q*, we next analyze the structure of $Cut(N')$ (see Fig. 5ii). Observe first that since $|Cut(N')| \ge 3$, the root of $N'$ must be contained in a gall $B'$ of $N'$. Let $h'\in V(N')$ denote the unique hybrid vertex of $B'$. Let $C_p,C_q\in Cut(N')$ be such that $p\in C_p$ and $q\in C_q$.

We claim that $C_p$ and $C_q$ are distinct elements in $Cut(N')-\{C_x,C_y,C_z\}$. To see this, note first that, since the sets $C_x$, $C_y$ and $C_z$ are pairwise distinct and $t,t'\in \mathcal R(N')$, it follows that one of *x*, *y*, and *z* must be contained in $C_{N'}(h')$. Without loss of generality, assume that $z\in C_{N'}(h') = C_z$. Note next that $C_p \ne C_q$. Indeed, at least two elements of $\{x,y,z\}$ are not contained in $C_p$, because $C_x, C_y$ and $C_z$ are distinct. Suppose, without loss of generality, $x\not \in C_p$. If $C_p = C_q$, then only the triplet *pq*|*x* will be contained in $\mathcal {R}(N')$, contradicting the fact that $t_1$ and $t_2$ are distinct triplets on $\{x,p,q\}$ contained in $\mathcal {R}(N')$. It remains to show that $C_p,C_q\not \in \{C_x,C_y,C_z\}$. Assume for the sake of contradiction that $p\in C_x$. Then only *xp*|*q* is in $\mathcal {R}(N')$, because $q \not \in C_x$, contradicting the fact that both $t_1$ and $t_2$ are in $\mathcal {R}(N')$. Similarly, if *p* is in $C_y$, then at most one of the two triplets on $\{y,p,q\}$ are in $\mathcal {R}(N')$, and if *p* is in $C_z$, at most one of the two triplets on $\{z,p,q\}$ are in $\mathcal {R}(N')$. So $C_p \not \in \{C_x, C_y, C_z\}$. By a symmetrical argument, $C_q \not \in \{C_x, C_y, C_z\}$. This proves the claim.

By the previous claim, neither *p* nor *q* is in $C_z$. Since $x\not \in C_z$ it follows that $t_1$ and $t_2$ cannot both be contained in $\mathcal {R}(N')$ which gives the final contradiction. Thus, $Cut(N)\subseteq Cut(N')$, as required.

Since both *Cut*(*N*) and $Cut(N')$ are partitions of *X*, it follows that $Cut(N)=Cut(N')$. $\square$

## Triplet systems that ${\mathcal L}_1(X)$-define

**Fig. 6:**
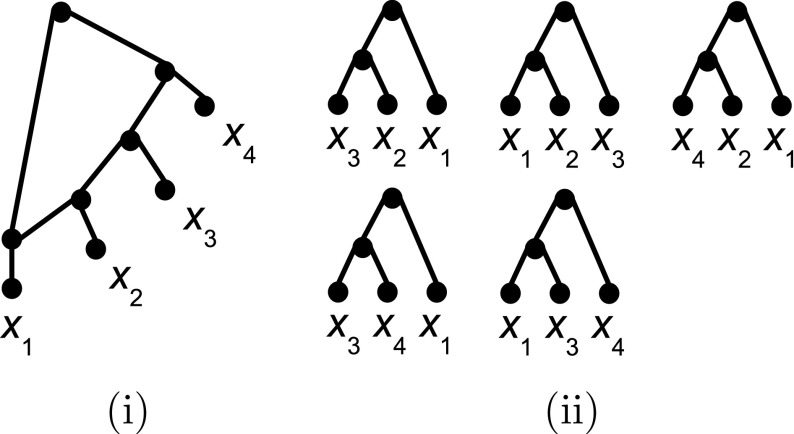
The binary level-1 *N* on $X=\{x_1,\ldots , x_4\}$ depicted in (**i**) is uniquely determined by the five triplets pictured in (**ii**) but $|\mathcal R(N)|=7$

As is well-known, every binary phylogenetic tree *T* on *X* is defined by the triplet set $\mathcal R(T)$ induced by *T*, where a a phylogenetic tree *S* on *X* is said to be *defined* by a triplet system $\mathcal R$ on *X*, if, up to equivalence, *S* is the unique phylogenetic tree on *X* for which $\mathcal R\subseteq \mathcal R(S)$ holds [see e. g. (Semple and Steel 2003)]. In this context it is important to note that this uniqueness only holds within the space of phylogenetic trees because all networks $N\in {\mathcal L}_1(X)$ that display *T* have the property that $\mathcal R(T) \subseteq \mathcal R(N)$. Combined with the fact that the network *N* pictured in Fig. 6i is, up to equivalence, the only binary level-1 network on $X=\{x_1,\ldots , x_4\}$ that is consistent with all five triplets depicted in Fig. 6ii—a simple case analysis can be applied to verify this—and $|\mathcal R(N)|= 7$, it is natural to ask how many triplets suffice to “uniquely determine” a level-1 network. In this section we provide a partial answer to this question. More precisely, saying that a network $N\in {\mathcal L}_1(X)$ is ${\mathcal L}_1(X)$ *-defined by a triplet system* $\mathcal R$ *(on* *X*) if, up to equivalence, *N* is the unique network in ${\mathcal L}_1(X)$ such that $\mathcal R\subseteq \mathcal R(N)$ holds, we show that every 4-outwards network *N* in ${\mathcal L}_1(X)$ that is also simple is ${\mathcal L}_1(X)$-defined by a triplet system of size at most $2|X|-1$. In addition, we show that if the requirement that *N* is simple is replaced by the requirement that *N* is saturated, then *N* is ${\mathcal L}_1(X)$-defined by $\mathcal R(N)$.

We note that 4-outwards is certainly a necessary condition for a network in $N\in {\mathcal L}_1(X)$ to be ${\mathcal L}_1(X)$-defined by any triplet system. In particular, if a network *N* has a gall with exactly three outgoing cut arcs then these can be permuted without affecting $\mathcal {R}(N)$. However, we shall see later that 4-outwards is not, in isolation, a sufficient condition.

As the next result shows not all triplets in $\mathcal R(N)$ are required to ${\mathcal L}_1(X)$-define a network $N\in {\mathcal L}_1(X)$ in case *N* is not only 4-outwards but also simple. To simplify its exposition, we say that a triplet system on *X* ${\mathcal L}_1(X)$ *-defines* a network $N\in {\mathcal L}_1(X)$ if *N* is ${\mathcal L}_1(X)$ *-defined* by it.

### Theorem 6.1

Every simple network in ${\mathcal L}_1(X)$ with at least four leaves is ${\mathcal L}_1(X)$-defined by a triplet system of size at most $2|X|-1$.

### Proof

We prove the theorem by induction on $|X|\ge 4$. Suppose *N* is a simple network in ${\mathcal L}_1(X)$, where $n=|X|\ge 4$. Let $X=\{x_1,\ldots , x_n\}$. Assume without loss of generality that $x_1$ is the head of the outgoing arc of the unique gall *C* of *N* starting at the hybrid vertex $v_1$ of *C*. If $n=4$ then a straightforward case analysis implies that *N* is ${\mathcal L}_1(X)$-defined by $\mathcal R(N)$. Note that $|\mathcal R(N)|=7=2n-1$ holds in this case.

Now assume that $n\ge 5$ and that for every set *Y* with $4 \le |Y|\le n-1$ and every simple network $N'\in {\mathcal L}_1(Y)$ there exists a triplet system $\mathcal R$ of size at most $2|Y|-1$ such that $N'$ is ${\mathcal L}_1(Y)$-defined by it. Starting at $v_1$ and traversing the unique cycle *C* in the underlying graph *U*(*N*) of *N* counter-clockwise let $v_1,v_2,\ldots , v_{i-1},v_i=\rho _N, v_{i+1},\ldots , v_{n+1},v_1$ denote a circular ordering of the vertices of *C*. Without loss of generality assume that for all $2\le j\le i-1$ the head of the outgoing arc of *C* starting at $v_i$ is $x_i$ and that for all $i+1\le j\le n+1$ the head of the outgoing arc of *C* starting at $v_j$ is $x_{j-1}$. Let $X'=X-\{x_n\}$. We distinguish between the cases that (i) the root $\rho _N$ of *N* equals $v_{n+1}$ and (ii) that this is not the case.

Case (i): Assume that $\rho _N=v_{n+1}$ and let $N'=N|_{X'}$. Since $N'$ is clearly simple and $4\le |L(N')|= n-1$ it follows by the induction hypothesis that there exists a triplet system $\mathcal R'$ on $X'$ of size at most $2(n-1)-1$ such that $N'$ is ${\mathcal L}_1(X')$-defined by $\mathcal R'$. Let $t_1=x_1|x_{n-1}x_n$ and $t_2=x_n|x_1x_{n-1}$. We claim that *N* is ${\mathcal L}_1(X)$-defined by $\mathcal R=\mathcal R'\cup \{t_1,t_2\}$. To see this, assume that $N_1$ is a network in ${\mathcal L}_1(X)$ for which $\mathcal R\subseteq \mathcal R(N_1)$ holds. We need to show that *N* and $N_1$ are equivalent.

Let $N_1'=N_1|_{X'}$. By construction, $\mathcal R'\subseteq \mathcal R(N_1')$. Since $N'$ is ${\mathcal L}_1(X')$-defined by $\mathcal R'$ it follows that $N'$ and $N_1'$ must be equivalent. Consequently $N_1'$ must also be a simple network in ${\mathcal L}_1(X')$. Combined with the fact that $t_1,t_2\in \mathcal R\subseteq \mathcal R(N_1)$ it follows that *N* and $N_1$ must be equivalent. Indeed, let *w* denote the parent of $x_n$ in $N_1$. Then $t_2$ implies that *w* is either the head of one of the two arcs of $N_1$ starting at the root $\rho '$ of the unique cycle of $N_1$ or a child of the root of $N_1$. Since $t_1$ implies that the parent $w'$ of $x_{n-1}$ is below *w*, it follows that *w* must lie on the path in $N_1$ from $\rho '$ to $w'$.

Case (ii) Assume that $\rho _N\not = v_{n+1}$. Then $i\in \{2,\ldots ,n\}$. We distinguish between the cases that $i=n$, that is, $\rho _N=v_n$ and that $i\in \{2,\ldots ,v_{n-1}\}$. In the former case the proof of the induction step is similar to the previous case but with $t_1$ replaced by $x_{n-1}|x_nx_1$. In the latter case the proof of that step is also similar to the previous case but this time with $t_2$ replaced by $x_{n-1}|x_nx_1$. $\square$

Combined with the definition of ${\mathcal L}_1(X)$-defining triplet systems, Theorem 6.1 immediately implies:

### Corollary 6.2

Every simple network in ${\mathcal L}_1(X)$ with at least four leaves is ${\mathcal L}_1(X)$-defined by its induced triplet system.

An obvious problem with extending Corollary 6.2 to general networks in ${\mathcal L}_1(X)$ is that 4-outwards networks can have tree-like regions. Consider, for example, then situation when a a 4-outwards network *N* contains a directed path of length 3 or more consisting solely of cut arcs. We can then transform it into a new network $N'$ in ${\mathcal L}_1(X)$ for which $\mathcal R(N)\subseteq \mathcal R(N')$ holds by subdividing the first and last cut arc of that path by new vertices *u* and *v*, respectively, and adding a new arc (*u*, *v*). There are however more subtle situations possible which do not require adding vertices and arcs. Consider, for example, the two networks *N* and $N'$ on $X=\{x_1,\ldots , x_5\}$ presented in Figures 2ii and 3ii, respectively. Then $\mathcal R(N')\subseteq \mathcal R(N)$ holds but *N* and $N'$ are clearly not equivalent. Thus, $N'$ is not ${\mathcal L}_1(X)$-defined by $\mathcal R(N')$ (although $N'$ is clearly encoded by $\mathcal R(N')$ as it is 4-outwards Gambette and Huber 2012). We therefore next turn our attention to identifying additional conditions which allow 4-outwards networks to be ${\mathcal L}_1(X)$-defined by their induced triplet systems.

To establish our next result (Theorem 6.3), we require a construction from Iersel and Kelk (2011) that allows us to associate a level-1 network *Collapse*(*N*) to any level-1 network *N* such that *Collapse*(*N*) is either simple, or is a phylogenetic tree on two leaves. We next review this construction for networks in ${\mathcal L}_1(X)$.

Let *N* be a network in ${\mathcal L}_1(X)$. For each element $C\in Cut(N)$ choose some element $c_C\in C$ and let $X^*=\{c_C\,:\,C\in Cut(N)\}$. Note that $|X^*|\ge 2$, but if *N* is saturated $|X^*|\ge 3$ and if *N* is saturated and 4-outwards $|X^*|\ge 4$. Then the rooted DAG *Collapse*(*N*) is obtained from *N* as follows: for each highest cut arc (*u*, *v*) of *N*, replace the (directed) subgraph of *N* containing *v* and all vertices below *v* (we denote this subgraph $N_{x_v}$ for later use) by the unique element $x_v$ in $X^*\cap C_N(v)$. Clearly, if $|C_N(v)|\ge 2$ then $N_{x_v}$ is contained in ${\mathcal L}_1(C_N(v))$ and is an isolated vertex otherwise. That *Collapse*(*N*) is a simple network in ${\mathcal L}_1(X^*)$ or a phylogenetic tree on two leaves is clear. Let $\mathcal R_{Collapse(N)}$ denote the triplet system on $X^*$ comprising all triplets $x_w | x_u x_v$ for which there exist $x_1 \in C_N(w)$, $x_2 \in C_N(u)$ and $x_3 \in C_N(v)$ such that $x_1|x_2x_3\in \mathcal R(N)$. It is straightforward to see that $\mathcal R(Collapse(N)) = \mathcal R_{Collapse(N)}$.

### Theorem 6.3

Every 4-outwards network in ${\mathcal L}_1(X)$ that is also saturated is ${\mathcal L}_1(X)$-defined by its induced triplet system.

### Proof

We prove the theorem by induction on the number *g*(*N*) of galls in a saturated, 4-outwards network $N\in {\mathcal L}_1(X)$. Suppose *N* is such a network. Let $g=g(N)$. Then since $|X|\ge 2$ and *N* is saturated we have $g\ge 1$. Hence, $|X|\ge 3$. In case $g=1$, the assumption that *N* is saturated implies that *N* is simple, and thus $|X| \ge 4$ because *N* is 4-outwards. By Corollary 6.2, *N* is ${\mathcal L}_1(X)$-defined by $\mathcal R(N)$.

So assume that $g\ge 2$ and that every saturated, 4-outwards network $N\in {\mathcal L}_1(Y)$ with $g-1$ galls is ${\mathcal L}_1(Y)$-defined by a triplet system on *Y*, where *Y* is a finite set of size at least two. Let $N'\in {\mathcal L}_1(X)$ denote a network for which $\mathcal R(N) \subseteq \mathcal R(N')$ holds. We need to show that *N* and $N'$ are equivalent. To see this, we first analyze the networks *Collapse*(*N*) and $Collapse(N')$.

Note first that, by Proposition 5.1, $Cut(N') \,{=}\,Cut(N)$. Hence, we may assume without loss of generality that $X^*$ is the leaf set of both *Collapse*(*N*) and $Collapse(N')$. Next note that *Collapse*(*N*) is 4-outwards because *N* is 4-outwards and $|Cut(N)|\ge 4$. Since a simple level-1 network is in particular saturated and *Collapse*(*N*) has precisely one gall, the base case of the induction implies that *Collapse*(*N*) is ${\mathcal L}(X^*)$-defined by $\mathcal R_1:=\mathcal R(Collapse(N))$. Since with $\mathcal R_2:=\mathcal R(Collapse(N'))$ we have $\mathcal R_{Collapse(N)}=\mathcal R_1 \subseteq \mathcal R_2= \mathcal R_{Collapse(N')}$ and so $\mathcal R_1\subseteq \mathcal R_2$ holds it follows that *Collapse*(*N*) and $Collapse (N')$ must be equivalent.

We next analyze the level-1 networks $N_v$ of *N* with $v\in X^*$. Let $v\in X^*$ and let $C\in Cut(N)$ be such that $v\in C$. Note that if $|C|=1$ then $N_v$ is an isolated vertex and thus a rooted DAG with leaf set $\{v\}$. So assume that $|C|\ge 2$. Then since *N* is a saturated, 4-outwards network in ${\mathcal L}_1(X)$, $N_v$ is a saturated, 4-outwards network in ${\mathcal L}(C)$. Since $N_v$ has at most $g-1$ galls the induction hypothesis implies that $N_v$ is ${\mathcal L}(C)$-defined by $\mathcal R(N_v)$. By assumption, $\mathcal R(N) \subseteq \mathcal R(N')$ and so $\mathcal R(N_v) \subseteq \mathcal R(N'_v)$. Thus $N'_v$ and $N_v$ must be equivalent. Combined with the observation that the networks *Collapse*(*N*) and $Collapse (N')$ are equivalent it follows that *N* and $N'$ are equivalent. $\square$

## ${\mathcal L}_1(X)$-defining cluster systems

In this section, we turn our attention to the companion question of Sect. 6. That is, whether some (not necessarily proper) subset of $\mathcal S(N)$ is sufficient to “uniquely determine” a 4-outwards network *N* in ${\mathcal L}_1(X)$. We first present a formalization of the idea of “uniquely determining” to being ${\mathcal L}_1(X)$-defined for cluster systems. Subsequent to this, we then show that all 4-outwards networks $N\in {\mathcal L}_1(X)$ that are also simple are ${\mathcal L}_1(X)$-defined by a cluster system of size at most |*X*| (Theorem 7.1 and Corollary 7.2). Replacing the requirement that *N* is simple by the more general requirement that *N* is saturated, we also show that such networks are ${\mathcal L}_1(X)$-defined by their induced softwired cluster system (Theorem 7.3).

Let *N* denote a phylogenetic network on *X* and let $\mathcal S$ denote a cluster system on *X*. Then we say that *N* *displays* $\mathcal S$ *(in the softwired sense)* if $\mathcal S\subseteq \mathcal S(N)$ holds. Furthermore, we say that a network $N\in {\mathcal L}_1(X)$ is ${\mathcal L}_1(X)$ *-defined by a cluster system* $\mathcal S$ *on* *X* if, up to equivalence, *N* is the unique network in ${\mathcal L}_1(X)$ that displays $\mathcal S$. It should be noted that, as in the case of triplet systems, a binary phylogenetic tree *T* on *X* is not ${\mathcal L}_1(X)$-defined by its induced cluster system $\mathcal C(T)=\mathcal S(T)$. The reason is again that, by subdividing arcs of *T* and adding new arcs joining the subdivision vertices, we can transform *T* into a network *N* in ${\mathcal L}_1(X)$ for which $\mathcal C(T)\subseteq \mathcal S(N)$ holds. Also it should be noted that a network in ${\mathcal L}_1(X)$ is not ${\mathcal L}_1(X)$-defined by its induced hardwired cluster system. Analogous to the triplet result presented in Sect. 6, a 4-outwards networks $N\in {\mathcal L}_1(X)$ also need not be ${\mathcal L}_1(X)$-defined by $\mathcal S(N)$. Indeed, consider again the two 4-outwards networks $N_1$ and $N_2$ on $X=\{x_1,\ldots , x_5\}$ presented in Figures 3i and 2i, respectively. Then $N_1$ and $N_2$ are clearly not equivalent but $\mathcal S(N_1) \subseteq \mathcal S(N_2)$.

### Theorem 7.1

Let $X=\{x_1,\ldots , x_n\}$, $n\ge 4$, and suppose that *N* is a simple network in ${\mathcal L}_1(X)$ such that, when starting at the hybrid vertex $v_1$ of *N* and traversing the unique cycle *C* of *U*(*N*) counter-clockwise, the obtained vertex ordering for *C* is $v_1, v_2, \ldots , v_{i-1}, v_i=\rho _N, v_{i+1},\ldots , v_{n+1}, v_1$ and $x_j$ is a child of $v_j$, for all $1\le j\le i-1$, and $x_j$ is a child of $v_{j+1}$, for all $i\le j \le n$. Assume without loss of generality that $i-2\ge n-i+1$ i.e. that the right side of the gall contains at least as many leaves as the left side. Then *N* is ${\mathcal L}_1(X)$-defined by the cluster system $\mathcal S_d(X)$ where

(i) $$\mathcal S_d(N):=\bigcup _{2\le j\le n-1}\{\{x_1,x_2,\ldots ,x_j\}\} \cup \{ \{x_2,x_3,\ldots , x_n\}\}$$ if $$\rho _N=v_{n+1}$$.
(ii) $$\mathcal S_d(N):= \bigcup _{3\le j\le n-1}\{\{x_2,x_3\ldots , x_j\}\} \cup \{\{x_1,x_2\},\{x_1,x_n\},\{x_1,x_2,x_3\}\}$$ if $$\rho _N=v_n$$.
(iii) $$\mathcal S_d(N):=\bigcup _{3\le j\le i-1}\{\{x_2,x_3\ldots , x_j\}\}\cup \bigcup _{n-1 \ge j\ge i}\{\{x_n,x_{n-1},\ldots , x_j\}\}$$ $$\qquad \cup \{\{x_1,x_2\},\{x_1,x_n\},\{x_1,x_n,x_{n-1}\}\}$$ if $$\rho _N\not \in \{v_n,v_{n+1}\}$$.

### Proof

Let $N_1 \in \mathcal {L}_1(X)$ be a network such that $\mathcal {S}_d(N) \subseteq \mathcal {S}(N_1)$. We first claim that $N_1$ must be simple. Assume for the sake of contradiction that $N_1$ is not simple, that is, $N_1$ contains a non-trivial cut arc (*u*, *v*). Then every cluster in $\mathcal {S}(N_1)$ must be compatible with $C= C_{N_1}(v)$, $2 \le |C| < n$, and $C\in \mathcal {S}(N_1)$. We will derive a contradiction by showing that $\mathcal {S}_d(N)$, and thus also $\mathcal {S}(N_1)$, contains at least one cluster that is incompatible with *C*.

Case (i). We distinguish between the two alternatives that $x_1 \in C$ and that $x_1 \not \in C$. Assume first that $x_1 \in C$. Then since $2 \le |C| < n$ we have for $C':=\{x_2, \ldots , x_n\}\in \mathcal {S}_d(N)$ that $C' \cap C\not =\emptyset$ and that $C' \cap (X\backslash C) \not =\emptyset$, that is, $C'\not \subseteq C$. Since $x_1\in C$ it follows that $C\subseteq C'$ cannot hold either and so *C* and $C'$ are incompatible, as required. Now, suppose $x_1 \not \in C$. Then since $2 \le |C|$ there exist $p,q\in \{2,\ldots , n\}$ with $p<q$, say, such that $x_p, x_q \in C$. Clearly, $x_q\not \in C':= \{x_1, \ldots , x_p\}\in \mathcal {S}_d(N)$. But then $C'$ and *C* are again incompatible, as required.

A similar analysis holds for cases (ii) and (iii); we leave the details to the interested reader. Hence, $N_1$ must be simple, as claimed.

Let *h* denote the unique hybrid vertex of $N_1$ and let *x* denote the leaf of $N_1$ that is incident with *h*. For the remainder of the proof, we consider each of the three cases stated in the theorem separately. All three cases use the following observations: (a) if $N_1 |_{X-x}$ is a tree, then all clusters in $\mathcal {S}( N_1 |_{X-x} )$ are pairwise compatible; (b) If $\mathcal {S}_d(N)\subseteq \mathcal {S}(N_1)$, then $\mathcal {S}_d(N)|_{X-x}\subseteq \mathcal {S}(N_1)|_{X-x}$ where for any cluster system $\mathcal C$ of *X* we let $\mathcal C|_{X-x} = \{ C{\setminus }\{x\}\, :\,C \in \mathcal C\}$; (c) $\mathcal {S}(N_1 |_{X-x}) = \mathcal {S}(N_1)|_{X-x}$. For ease of presentation we will liberally make use of the assumption that $\mathcal {S}_d(N) \subseteq \mathcal {S}(N_1)$ without explicitly stating it.

Case (i). First, we argue that $x \in \{x_1, x_2\}$. Assume for the sake of contradiction that $x \not \in \{x_1, x_2\}$. Then $C = \{x_1, x_2\}$ and $C' = \{x_2, \ldots , x_n\}{\setminus }\{x\}$ are incompatible and clearly contained in $\mathcal {S}_d(N)|_{X-x}\subseteq \mathcal {S}(N_1)|_{X-x}$. Hence, $\mathcal {S}(N_1)|_{X-x}$ is not compatible which is impossible because *x* is incident with *h* and so $N_1 |_{X-x}$ is a phylogenetic tree. So $x \in \{x_1, x_2\}$. In fact, similar reasoning implies that $x = x_2$ is also impossible as otherwise $\mathcal {S}_d(N)|_{X-x}$ would contain incompatible clusters $\{x_1, x_3\}$ and $\{x_3, \ldots , x_n\}$. So $x=x_1$. Since $\{x_1, x_2\} \subseteq \mathcal {S}_d(N)$ it follows that the other child of the parent of $x_2$ in $N_1$ is *h*. Combined with the fact that $\bigcup _{2 \le j \le n-1} \{ \{x_1, x_2, \ldots , x_j\}\} \subseteq \mathcal {S}_d(N)$ it follows that the other child of the parent of $x_k$ in $N_1$ is the parent of $x_{k-1}$, $3 \le k \le n-1$. Since $\{x_2, \ldots , x_n\} \in \mathcal {S}_d(N)$ it follows that the other child of the parent of $x_n$ in $N_1$ is the parent of $x_{n-1}$. Hence $N_1$ is equivalent to *N*.

Case (ii). We claim that $x \in \{x_1, x_2, x_n\}$. The argument is similar to case (i) in that if $x \not \in \{x_1, x_2, x_n\}$ then $\mathcal {S}_d(N)|_{X-x}\subseteq \mathcal {S}(N_1)|_{X-x}$ contains incompatible clusters $\{x_1, x_2\}$ and $\{x_1, x_n\}$, leading to a contradiction of the fact that $N_1 |_{X-x}$ is a phylogenetic tree. In fact, similar arguments utilizing the facts that $\{x_1, x_2, x_3 \} \in \mathcal {S}_d(N)$ and that $n \ne 3$ imply that $x \ne x_2$ and $x \ne x_n$. So again $x = x_1$. Since $\{x_1, x_2\}$ and $\{x_1, x_n\}$ are contained in $\mathcal S_d(N)\subseteq \mathcal S(N_1)$ it follows that the other child of the parents of $x_2$ and $x_n$ in $N_1$, respectively, is *h*. In view of $\{x_1,x_2,x_3\} \subseteq \mathcal S_d(N) \subseteq \mathcal S(N_1)$ we see that the other child of the parent of $x_3$ in $N_1$ must be the parent of $x_2$. Since $\bigcup _{3\le j\le n-1}\{\{x_2,x_3\ldots , x_j\}\} \subseteq \mathcal S_d(N)$ similar arguments as in the previous case imply that *N* and $N_1$ must be equivalent.

Case (iii). Again the fact that $N_1 |_{X-x}$ is a phylogenetic tree implies that $x \in \{x_1, x_2, x_n\}$. However, $x = x_n$ cannot hold because $n-1 \ne 2$ and so $\{x_1, x_2\}$ and $\{x_1, x_{n-1}\}$ are distinct clusters that are both contained in $\mathcal {S}_d(N)|_{X-x}$ and thus in $\mathcal {S}(N_1)|_{X-x}$. But then $\mathcal {S}(N_1)|_{X-x}$ is incompatible which is impossible as $N_1 |_{X-x}$ is a phylogenetic tree. Similarly, $x \not = x_2$ as otherwise the two incompatible clusters $\{x_1, x_n\}$ and $\{x_n, x_{n-1}, \ldots , x_i\}$ are contained in $\mathcal {S}_d(N)|_{X-x}$. So $x = x_1$. Focussing as in case (ii) on $x_2$ and $x_n$ we see again that the common child of their respective parents is *h*. Since $\bigcup _{3\le j\le i-1}\{\{x_2,x_3\ldots , x_j\}\}\cup \bigcup _{n-1 \ge j\ge i}\{\{x_n,x_{n-1},\ldots , x_j\}\} \subseteq \mathcal S_d(N)$ the location of the remaining leaves of $N_1$ is forced. Hence, $N_1$ is equivalent to *N*. $\square$

As an immediate consequence of Theorem 7.1, we obtain the companion result for Theorem 6.1.

### Corollary 7.2

Every simple network in ${\mathcal L}_1(X)$ with at least four leaves is ${\mathcal L}_1(X)$-defined by a cluster system of size at most |*X*|.

We now prove the cluster equivalent of Theorem 6.3 i. e. that requiring that a 4-outwards network in ${\mathcal L}_1(X)$ is also saturated guarantees that it is uniquely determined by its induced softwired cluster system.

### Theorem 7.3

Every 4-outwards network in ${\mathcal L}_1(X)$ that is also saturated is ${\mathcal L}_1(X)$-defined by its induced softwired cluster system.

### Proof

Let *N* and $N'$ be networks in ${\mathcal L}_1(X)$ such that *N* is 4-outwards and saturated and $\mathcal S(N) \subseteq \mathcal S(N')$ holds. We need to show that $N'$ is equivalent with *N*. Let $\mathcal T = \mathcal T(N)$. Clearly, $\bigcup _{T \in \mathcal T} \mathcal S(T) = \mathcal S(N)\subseteq \mathcal S(N')$. Combined with (Iersel and Kelk 2011, Proposition 1) which implies that $\mathcal R(\mathcal T) \subseteq \mathcal R(N')$ and the fact that $\mathcal R(N) = \mathcal R(\mathcal {T})$ it follows that $\mathcal R(N) \subseteq \mathcal R(N')$. Since, by Theorem 6.3, *N* is ${\mathcal L}_1(X)$-defined by $\mathcal R(N)$ it follows that *N* and $N'$ are equivalent. $\square$

In fact, due to the very general character of (Iersel and Kelk 2011, Proposition1) Theorem 7.3 can easily be extended to prove that, whenever $\mathcal R(N)$ has been proven sufficient to uniquely determine (in our sense) a specified subfamily—any subfamily—of phylogenetic networks *N*, so too is $\mathcal S(N)$ where we canonically extend the notions of an induced triplet system and softwired cluster system to such networks.

## Conclusions

In this paper, we have presented enumerative results concerning the number of vertices, arcs, and galls of a binary level-1 network. By focusing on triplet systems and (softwired) cluster systems we have also investigated the question if subsets of those systems suffice to uniquely determine the binary level-1 network that induced them. As part of this, we have presented examples that illustrate that a level-1 network need not be uniquely determined by the triplet/cluster system it induces, thus illustrating the difference between the notion of encoding and our formalization of uniquely determining. In addition, we have provided bounds on the size of such a system in case the network in question is simple and has at least four leaves. For the more general class of 4-outwards, saturated, binary level-1 networks we have shown that any network in that class is uniquely determined by the triplet/softwired cluster system it induces. However, a number of open questions remain. For example for which binary level-1 networks are the aforementioned bounds sharp and are 4-outwards saturated binary level-1 networks characterizable by the fact that they are uniquely determined by their induced triplet/softwired cluster system?

We conclude with remarking that in Huber and Moulton (2013) *trinets*, that is, rooted directed acyclic graphs on just three leaves have recently been introduced in the literature for phylogenetic network reconstruction. In that paper it was also shown that any level-1 network is encoded by the trinet system that it induces. In addition, it was shown in Iersel and Moulton (2014) that the more general tree-sibling and level-2 networks are encoded by their induced trinet systems, a fact that is not shared in general for the triplet system or the softwired cluster system induced by such networks. Formalizing the idea of “uniquely determining” for trinet systems in a canonical way to ${\mathcal L}_1(X)$ *-defining trinet systems* it might be interesting to explore what kind of trinet systems ${\mathcal L}_1(X)$-define such networks.

## Footnotes

1 In this paper only a subset of these “cleaning up” operations will be required. However, we list them all to retain consistency with the wider literature.

2 There is some confusion in earlier literature whether $\emptyset$ should be considered an SN-set. Here we allow this, as it does not adversely affect our analysis. Although it sounds a little strange, $\emptyset$ is also a non-trivial SN-set. We adopt this convention to ensure consistency with earlier publications: *X* is the only trivial SN-set.
